# One-year implant survival following lateral window sinus augmentation using plasma rich in growth factors (PRGF): a retrospective study

**DOI:** 10.4317/medoral.23482

**Published:** 2020-03-06

**Authors:** Panagiotis Dragonas, Marlon Foote, Qingzhao Yu, Archontia Palaiologou, Pooja Maney

**Affiliations:** 1 DDS, MS. Department of Periodontics, School of Dentistry, Louisiana State University Health Sciences Center, New Orleans, LA; 2 DDS, MS. Private Practice, Orlando, FL.; 3 PhD. Biostatistics Program, School of Public Health, Louisiana State University Health Sciences Center, New Orleans, LA; 4 DDS, MS. Department of Periodontics, School of Dentistry, University of Texas Health San Antonio, San Antonio, TX; 5 BDS, MSD, PhD. Department of Periodontics, School of Dentistry, Louisiana State University Health Sciences Center, 1100 Florida avenue, New Orleans, LA

## Abstract

**Background:**

The aim of this study was to assess one-year implant survival after lateral window sinus augmentation using PRGF combined with various bone grafting materials.

**Material and Methods:**

This was a retrospective chart review and radiographic analysis of patients that had undergone lateral window sinus augmentation with PRGF and had dental implants placed at least 6 months post augmentation. All implants included were followed up for at least one year after placement. Demographic, sinus and implant related characteristics (residual ridge height, sinus membrane perforation, type of graft material, implant length and width and ISQ at placement) were analyzed.

**Results:**

A total of 31 patients with 39 sinus augmentations and 48 implants were included. The mean follow up was 22.8 ± 9.9 months. Implant survival was 95.8%, with 2 implants overall failing. Among all the variables assessed, the only one found to be associated with an increased risk for implant failure was the use of xenograft as bone grafting material in the sinus.

**Conclusions:**

Within the limitations of this study, dental implants placed in maxillary sinuses grafted with PRGF in combination with bone grafting materials, exhibit high implant survival rates after at least one year follow up.

** Key words:**PRGF, sinus graft, growth factors, implant survival, platelet concentrates.

## Introduction

Implant supported dental reconstruction in the posterior maxilla can be challenging, primarily due to insufficient alveolar ridge dimensions following loss of teeth and subsequent maxillary sinus pneumatization. Sinus floor augmentation, through the lateral window approach, has been successfully implemented to increase bone dimensions and allow for dental implant placement with high long-term survival rates (1). A variety of bone grafting materials have been successfully used for sinus augmentation including autogenous bone, xenografts, allografts and alloplasts. A recent study on bone quality outcomes following use of different grafting materials, reported that autogenous bone resulted in the highest amount of new bone formation and lowest amount of residual graft material compared to other options, whereas no difference was noted between allografts, xenografts and alloplasts (2). However, disadvantages associated with use of autogenous bone, including morbidity, limited availability and high volumetric changes (2), promoted the use of other biomaterials, often combined with platelet concentrates in an effort to enhance the regenerative outcome in maxillary sinus augmentation. Plasma rich in Growth factors (Endoret® PRGF) is an autologous blood derived product that was originally developed by Anitua *et al*. (3), in an effort to enhance wound healing and promote tissue regeneration when used in different intraoral bone grafting procedures. PRGF is based on a single centrifugation technique and requires the conjugation of anticoagulants with the freshly collected blood as well as the subsequent addition of calcium chloride in order to allow for the formation of a provisional adhesive matrix (4). Theoretically, the enmeshment of a supraphysiologic concentration of platelets within the matrix allows for the secretion of high concentration of growth factors including VEGF, PDGF and IGF which can enhance wound healing through stimulation of re-epithelialization, angiogenesis and extracellular matrix formation (5). In contrast to other platelet concentration technologies available, the PRGF preparation protocol aims at eliminating leukocytes in an effort to avoid any pro-inflammatory effects associated with them and allows for it to be used in diverse forms of clinically applicable products, such as clots, fibrin membrane and liquid, derived from the different fractions of the plasma column upon centrifugation(5). PRGF has been used in maxillary sinus augmentation in combination with various bone grafting materials with favorable outcomes in terms of new vital bone formation and post-operative swelling and pain (6-8). There is limited evidence though regarding survival rates of implants placed on maxillary sinuses grafted with PRGF (9). The aim of the present retrospective study was to assess implant survival with at least one year follow up after lateral window sinus augmentation using PRGF combined with various bone grafting materials.

## Material and Methods

- Study Design

This study was a retrospective chart review and analysis of corresponding cone beam computed tomography (CBCT) scans as well as periapical radiographs. The study protocol was approved by the Louisiana State University Health Sciences Center – New Orleans Institutional Review Board (IRB # 9727). Patients included in the study, were treated in the Department of Periodontics Postgraduate Clinic at LSUHSC School of Dentistry (SOD) from July 1st, 2010 to June 30th, 2016. Charts of patients along with Cone Beam Computed Tomography (CBCT) scans were carefully reviewed to identify individuals that conformed to the following inclusion criteria: 1) History of Lateral Window Sinus Augmentation with PRGF in combination with bone grafting materials; 2) CBCT scans available before and 6 months after sinus augmentation; 3) Dental implants placed in the grafted maxillary sinuses at least 6 months after augmentation and followed up radiographically for at least one year after implant placement.

**Figure 1:**
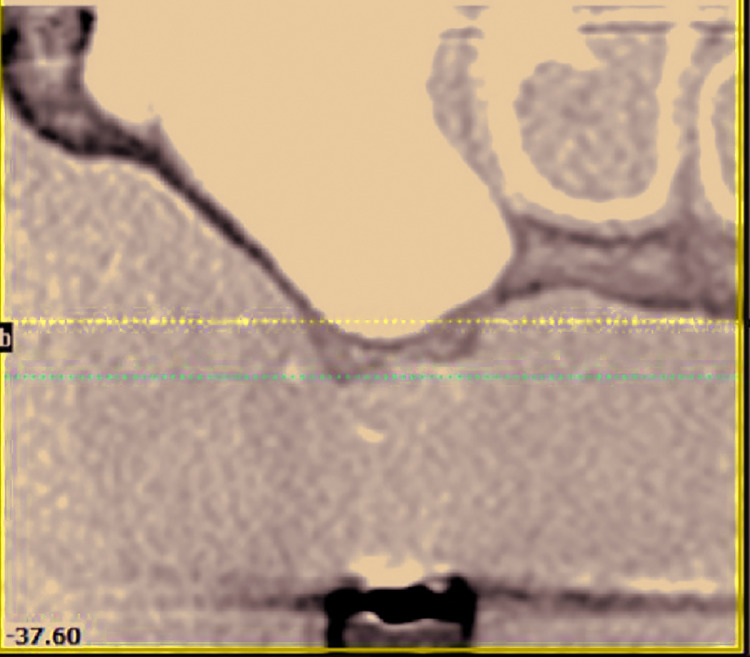
Lateral wall osteotomy with intact Schneiderian membrane.

All patients reviewed had previously undergone sinus floor augmentation surgery performed using a lateral window approach (10), after radiographic evaluation with a CBCT (Fig. 1).

**Figure 2:**
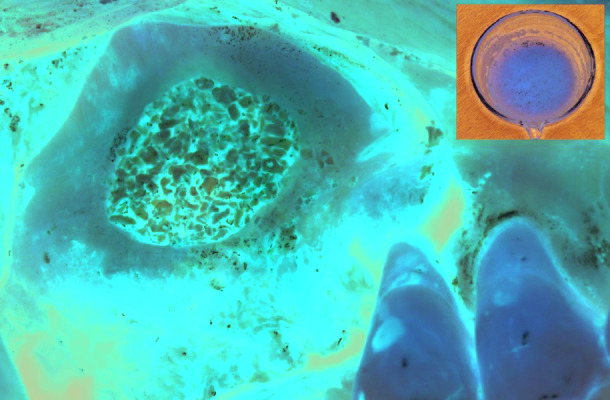
Placement of the xenograft mixed with PRGF into the sinus.

Each patient had undergone a blood draw for PRGF preparation as per the manufacturer’s protocol (PRGF®-Endoret® BTI Biotechnology Institute) (7). Briefly, blood was taken from patients by venipuncture before surgery into tubes containing 3.8% sodium citrate as anticoagulant. PRGF was prepared by centrifugation at 460g for 8 minutes at room temperature. Plasma not including the buffy coat above red blood cells, was pipetted out in two fractions. Fraction 1, closest to the red and white blood cell containing sediment, was mixed with calcium chloride on a dose dependent quantity and then mixed with a bone graft material (Fig. 2). The mix was incubated at 37C until a gel like consistency was formed. The product was then used as a graft material into the sinus cavity (Fig. 2).

**Figure 3:**
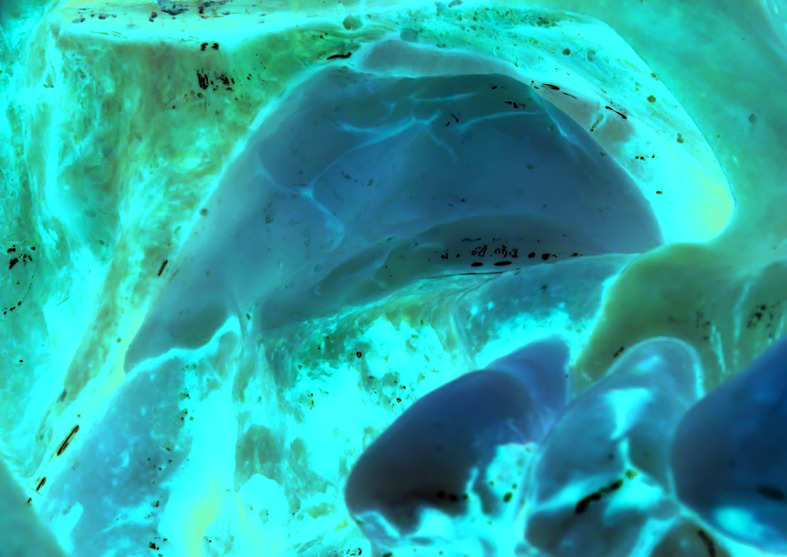
Lateral wall covered with PRGF membrane. The same PRGF membrane was used to seal intra-operative sinus membrane populations.

Fraction 2, immediately above fraction 1, was also mixed with calcium chloride on a dose dependent manner and the produced material was used as a membrane. The PRGF membrane was then placed over a collagen membrane covering the lateral window, prior to flap closure (Fig. 3).

When appropriate, PRGF membranes were also used to repair sinus membrane perforations. The grafting materials that were used for all sinus augmentations (in combination with PRGF) included either bovine anorganic bone (Bio-Oss®, Geistlich Biomaterials, Wolhusen, Switzerland), cancellous bone allograft (Puros®, Zimmer Biomet, Carlsbad, CA) or a combination of both graft materials. None of the patients had implant placed simultaneously with maxillary sinus augmentation. Post-surgically, all patients received antibiotic coverage along with appropriate analgesics, anti-inflammatory medications and Chlorhexidine Rinse 0.12%. All patients received new CBCT scans approximately six months post maxillary sinus augmentation, to assess bone height prior to implant placement. Dental implants were placed following a 1 stage or 2-stage protocol. Implant stability was recorded using Implant stability Quotient (ISQ). After a healing period of at least 3 months, second stage surgery was completed with another ISQ recording and the patient was subsequently referred back for continuation of restorative treatment.

- Data collection

The following information was collected for each patient: demographic data (gender and age) and smoking habits at the time of surgery. Surgery related parameters recorded included (i) type of graft material that was mixed with PRGF (xenograft, allograft or combination), (ii) type of implant placed (brand, length, width), (iii) implant stability measured with implant stability quotient (ISQ) at the time of implant placement and stage 2 surgery and (iv) sinus membrane perforation at the time of surgery (Yes/No).

- CBCT Scan Analysis

All CBCT images were previously obtained with an iCat Next Generation XYZ Slice View Tomograph (Imaging Sciences International LLC, Hatfield, PA). For all CBCT images, a limited field of view (FOV) of 4x4 cm, 8x8 cm, 10x16 cm was selected. CBCT images were evaluated by a single provider (M.F.) in axial, sagittal and coronal planes using the iCatVision software, version 1.8.1.10 (Imaging Sciences International LLC, Hatfield, PA). CBCT images were analyzed using a 13-inch MacBook Pro with Retina display with a resolution of 1,440x900 (Apple Inc.) The following variables were assessed pre-operatively.

a. Residual bone height (RBH): Distance between alveolar bone crest and inferior border of the sinus at the area of the least amount of residual bone.

b. Thickness of the sinus membrane: Distance between inferior border of the sinus and mucosal surface on the planned area of sinus augmentation.

c. Morphology of sinus membrane was evaluated and classified according to criteria modified from Soikkonen and Ainamo (11) : 1) healthy sinus membrane with no thickening (<2mm); 2) flat: shallow thickening without well-defined outlines; 3) semispherical: thickening with well-defined outlines rising in an angle of > 30 degrees from the floor of the walls of the sinus; 4) mucocele-like: complete opacification of the sinus; 5) mixed-flat and semispherical thickening.

d. Presence or absence of septae

e. Presence or absence of alveolar antral artery (anastomosis of posterior superior alveolar artery and infraorbital artery)

At 6 months after sinus augmentation surgery, bone height at the CBCT was measured again to assess bone gain, with an effort made to make the measurements at the same site where the pre-operative measurements were made.

- Implant survival and success

Periapical radiographs taken at the time of implant placement, implant uncovery, implant restoration and at least 1 year follow up post placement were reviewed by a single individual (M.F.). Misch criteria for success (12) were used to assess implant outcomes at follow-ups measuring for radiographic bone loss: I. Success (optimum health): <2 mm radiographic bone loss from initial surgery; II. Satisfactory survival: 2–4 mm radiographic bone loss; III. Compromised survival: Radiographic bone loss >4 mm (less than 1/2 of implant body); IV. Failure: Radiographic bone loss >1/2 length of implant. 

- Statistical analysis

The primary outcome was implant survival (a binary variable). A survival analysis was planned but not performed due to the very small number of events (implant failure). For the same reason, a logistic regression model that jointly considered all risk factors together could not be performed. To explore risk factors that are significantly related with the implant survival, we performed a two-sample t-test for each continuous risk factor (Age, ISQ and Residual ridge height) and a Fisher’s exact test for each binary or categorical factor (sex, smoking, Schneiderian membrane perforation, bone graft material, implant length, implant diameter and stage placement). The significance level was set at 0.05. All analyses were performed using SAS version 9.4. 

## Results

A total of 31 patients (13 male, 22 female) with a mean age of 61.92 ± 7.45 (range 42–73 y/o) were included with 29% of them being current smokers. A total of 39 maxillary sinus augmentations were completed, with a total of 48 implants placed. Table 1 shows the demographic and maxillary sinus related characteristics. In general, residual ridge height was 3.9mm ± 1.66 with a mean sinus membrane thickening of 1.09mm ± 1.81. 10% of the sinuses had septae as identified in the CBCT scans and the alveolar antral artery was noted in 4 cases (10%).

**Table 1:**
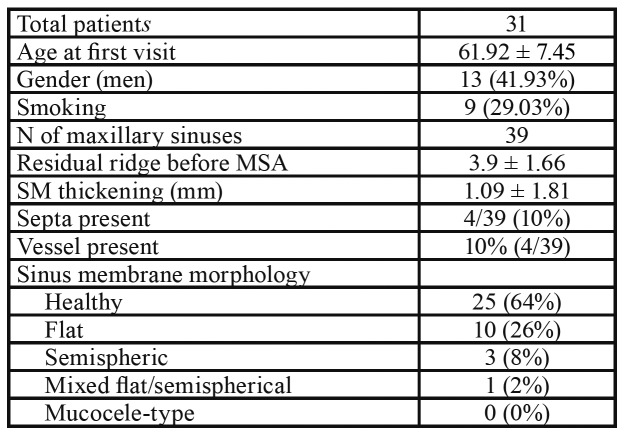
Distribution of demographic and maxillary sinus related characteristics.

The majority of the sinuses (69%) showed no thickening of the Schneiderian membrane, whereas 26% presented a flat shallow thickening with well-defined borders. During sinus augmentation, 36% of the sinuses had perforation of the Schneiderian membrane. Table 2 shows characteristics of the implants placed in the augmented sinuses.

**Table 2:**
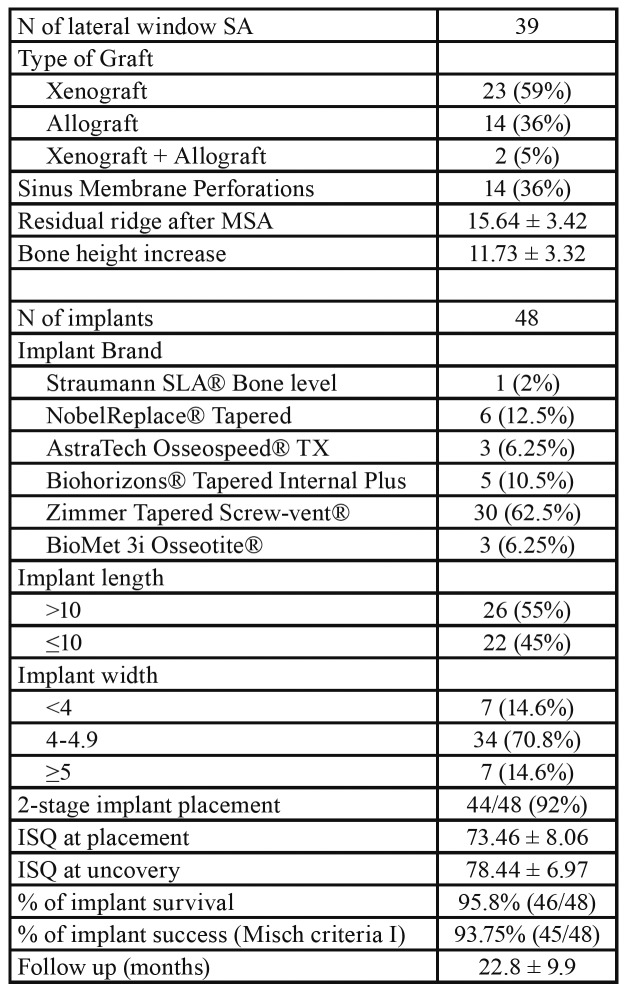
Distribution of MSA/Implant related characteristics.

Implants were followed up for a mean of 22.8±9.9 months. Overall, two implants failed (implant survival rate 95.8%), one of which as an early failure due to inability to establish osseointegration and the other as a late failure due to advanced peri-implantitis. Based on the one-year post-placement radiographic evaluation, implant success rate (Misch Criteria I) (12) was 93.75%. The majority of the implants placed were Zimmer Tapered Screw-vent® (62.5%) and the remaining were almost equally distributed among five other implant brands NobelReplace® Tapered, AstraTech Osseospeed® TX, Straumann SLA® Bone level, BioMet 3i Osseotite®, Biohorizons® Tapered Internal Plus. For the majority of the implants, an increase on ISQ measurements was noted from placement to stage 2 implant exposure, with an average increase from 73.46 ± 8.06 to 78.44 ± 6.97. Among all the variables assessed, the only one found to be associated with an increased risk for implant failure was the use of xenograft as bone grafting material in the sinus, either alone or in combination with an allograft (*p*=0.0275). None of the other factors including age, sex, smoking, ISQ at placement, residual ridge height, perforation of the sinus membrane, implant length, implant diameter or 1 vs. 2 stage implant placement were associated with implant survival (Table 3).

**Table 3:**
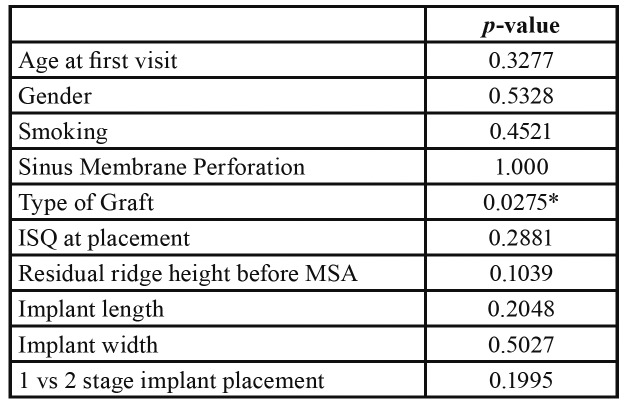
Association of patient characteristics with implant survival.

## Discussion

This retrospective study reports an implant survival rate of 95.8% for implants placed at least 6 months after maxillary sinus augmentation with bone graft plus PRGF and with at least one year follow up, in a university setting. This outcome is similar to survival rates of dental implants placed in grafted maxillary sinuses as reported in previously published systematic reviews (1,13). Specifically, Corbella *et al*. in a systematic review of 29 studies with at least 3 year implant follow up post lateral sinus augmentation using a variety of bone grafting materials, reported implant survival varying from 75.57% to 100% with the majority of the included studies reporting survival rates over 90% (13). In another systematic review Del Fabbro *et al*. reported implant survival of 93.7% after lateral sinus augmentation, with the majority of the failures occurring within the 1st year (1).

As a wide range of bone grafting materials have been used with good success in maxillary sinus augmentation procedures, the ideal grafting material is still debated in the literature. The amount of new bone formation (NBF) through histomorphometric analysis has been considered one important parameter to assess when evaluating success of maxillary sinus augmentation (14). Corbella *et al*. in a meta-analysis of histomorphometric outcomes following maxillary sinus augmentation reported that autogenous bone could lead to a higher percentage of new bone when compared to bovine bone (15). However, when considering non-comparative studies, they reported that it was not possible to detect any significant difference among examined biomaterials, postulating that in the presence of all conditions allowing for blood clot stability, the space below the Schneiderian membrane can become populated by osteoprogenitor cells that induce bone regeneration irrespective of the biomaterial used to fill the space (15). Studies have even advocated no use of bone substitutes in sinus floor elevations and allowing the site to fill in with only blood clot, with implants acting as a tent against the elevated Schneiderian membrane, reporting high implant survival rates (16).

Whether the selection of bone grafting material has any effect on dental implant survival has also been assessed. Nkenke *et al*. in a systematic review attempted to address whether the use of autogenous bone is superior to bovine bone in terms of implant survival and reported that the type of graft did not seem to be associated with implant survival rates and questioned the use of autogenous bone due to associated morbidity (17). In the present study, the only factor associated with implant failure was the use of xenograft as bone grafting material in the sinus. However, this finding contradicts current evidence reporting that the type of bone graft material in the sinus has no effect on implant survival (17). This finding may be attribuTable to the fact that the type of bone graft material used was not randomly assigned to the patients.

More recently, in an effort to enhance regenerative outcomes in maxillary sinus augmentation without the use of autogenous bone, bone grafting materials have been combined with autologous platelet concentrates, due to their high concentration of platelet derived growth factors. A variety of autologous preparation protocols have been introduced including Platelet-rich Plasma (PRP) (18), Platelet-rich Fibrin (PRF) (19) , and Plasma-rich in Growth factors (PRGF) (3), with a wide range of outcomes reported. Lemos *et al*. (20) in a systematic review, found no influence of PRP on bone formation and implant survival when used in maxillary sinus augmentation. Similarly, Dragonas *et al*. (21) in another systematic review, reported that the use of L-PRF in maxillary sinus augmentation was not associated with more favorable outcomes, in terms of new bone formation and soft tissue healing. With regards to the use of PRGF for maxillary sinus grafting, a recent review reported that its use was associated with positive outcomes for soft tissue healing and postoperative symptoms, however, the outcomes on new bone formation were conflicting, with one study reporting higher % of NBF in PRGF treated sites and two studies no difference in PRGF vs. control sites (22).

Overall however, there is limited evidence regarding implant survival in maxillary sinuses grafted with PRGF. Khouly *et al*. (9) reported 90% implant survival with a mean follow up of 7.2 years in 100 augmented sinuses with PRGF, with residual crestal bone of < 3mm and immediate loading being associated with increased risk for implant failure. Torres *et al*. (23) reported 98.6% of implant success after a 2 year follow up, in sites that were grafted with bovine bone and PRGF, with no difference when compared with implants placed in sinuses grafted with bovine bone alone. Similarly, Anitua *et al*. (7) reported 100% implant survival in sites grafted with PRGF after a mean follow up period of 33±7 months.

In the present study a high implant survival rate (95.8%) was noted despite the surgeries being completed by periodontics residents in training. According to a recent meta-analysis, surgical experience significantly affected implant failure rates (24). The high implant survival rate noted in this cohort could be attributed to a number of factors. First, all patients were assessed for presence of sinus pathologies through pre-surgical CBCT evaluation and referred for ENT consultation as needed. Prior to sinus augmentations, 90% of the patients radiographically presented with either sinus membrane with no thickening or flat shallow thickening with well-defined outlines. In 8% of our cases where semispheric sinus membrane morphology was noted (usually indicative of mucous retention cyst/antral pseudocyst), all were less than 5mm in height. Moreover, all implants were placed at least 6 months post sinus augmentation, with no cases of simultaneous implant placement, which has been reported as a potential risk factor, especially in the presence of minimal residual bone height (25). All implants also were placed with good primary stability as noted by the ISQ measurements taken at the time of implant placement (73.46 ± 8.06) which increased during the healing period (78.44 ± 6.97). ISQ values ranging from 57 to 82 denote appropriate implant stability and a complete process of osseointegration (26).

Perforation of the sinus membrane did not seem to be associated with implant failure in this study. A recent systematic review on 58 included studies reported that an intraoperative sinus membrane perforation could increase the risk of implant failure after sinus augmentation surgery (27). The authors commented that this positive relationship could be explained by the event of displacement of bone particles through the perforation, even in the event of membrane repair, resulting in chronic infection, bone graft resorption and reduction in bone formation (27). We cannot truly assess whether the use of PRGF as a membrane to repair sinus membrane perforation or mixed with the bone grafting material may have reduced complications associated with perforations in our study. However, the sticky consistency of the bone graft when mixed with PRGF may improve its graft-handling properties and help with reducing its displacement through the perforation (28). Also, the use of a PRGF membrane has been previously reported to assist in sealing membrane perforations during endodontic surgery as well as crestal and lateral sinus augmentations (8,29,30). Whether the use of PRGF contributed to the high implant survival rate noted in this study irrespective of the 36% incidence of membrane perforations cannot be assessed.

The study has some limitations due to the nature of it being a retrospective collection of data. First, the surgeries were completed by different clinicians under training which increases surgical variability and reduces treatment consistency. Also the number of individuals and sinus augmentations included in this analysis was relatively small, which did not allow us to perform a logistic regression analysis that could have simultaneously considered all risk factors.

## Conclusions

Within the limitations of this study, it can be concluded that dental implants placed in maxillary sinuses grafted with bone graft combined with PRGF, exhibited high implant survival rates after at least one-year follow up. The extent of PRGF contribution to the high implant survival rate though, needs to be assessed through randomized controlled trials, where the presence of controls (non-PRGF grafted sinuses) would further elucidate the effects of this blood-derived platelet concentrate.

## References

[1] Del Fabbro M, Wallace SS, Testori T (2013). Long-term implant survival in the grafted maxillary sinus: a systematic review. Int J Periodontics Restorative Dent.

[2] Danesh-Sani SA, Engebretson SP, Janal MN (2017). Histomorphometric results of different grafting materials and effect of healing time on bone maturation after sinus floor augmentation: a systematic review and meta-analysis. J Periodontal Res.

[3] Anitua E (1999). Plasma rich in growth factors: preliminary results of use in the preparation of future sites for implants. Int J Oral Maxillofac Implants.

[4] Anitua E, Alkhraisat MH, Orive G (2012). Perspectives and challenges in regenerative medicine using plasma rich in growth factors. J Control Release.

[5] Anitua E, Aguirre JJ, Algorta J, Ayerdi E, Cabezas AI, Orive G (2008). Effectiveness of autologous preparation rich in growth factors for the treatment of chronic cutaneous ulcers. J Biomed Mater Res B Appl Biomater.

[6] Anitua E, Prado R, Orive G (2012). Bilateral sinus elevation evaluating plasma rich in growth factors technology: a report of five cases. Clin Implant Dent Relat Res.

[7] Anitua E, Prado R, Orive G (2009). A lateral approach for sinus elevation using PRGF technology. Clin Implant Dent Relat Res.

[8] Del Fabbro M, Corbella S, Ceresoli V, Ceci C, Taschieri S (2015). Plasma Rich in Growth Factors Improves Patients' Postoperative Quality of Life in Maxillary Sinus Floor Augmentation: Preliminary Results of a Randomized Clinical Study. Clin Implant Dent Relat Res.

[9] Khouly I, Pardinas Lopez S, Aliaga I, Froum SJ (2017). Long-Term Implant Survival After 100 Maxillary Sinus Augmentations Using Plasma Rich in Growth Factors. Implant Dent.

[10] Tatum H Jr (1986). Maxillary and sinus implant reconstructions. Dent Clin North Am.

[11] Soikkonen K, Ainamo A (1995). Radiographic maxillary sinus findings in the elderly. Oral Surg Oral Med Oral Pathol Oral Radiol Endod.

[12] Misch CE, Perel ML, Wang HL, Sammartino G, Galindo-Moreno P, Trisi P (2008). Implant success, survival, and failure: the International Congress of Oral Implantologists (ICOI) Pisa Consensus Conference. Implant Dent.

[13] Corbella S, Taschieri S, Del Fabbro M (2015). Long-term outcomes for the treatment of atrophic posterior maxilla: a systematic review of literature. Clin Implant Dent Relat Res.

[14] Iwaniec UT, Wronski TJ, Turner RT (2008). Histological analysis of bone. Methods Mol Biol.

[15] Corbella S, Taschieri S, Weinstein R, Del Fabbro M (2016). Histomorphometric outcomes after lateral sinus floor elevation procedure: a systematic review of the literature and meta-analysis. Clin Oral Implants Res.

[16] Duan DH, Fu JH, Qi W, Du Y, Pan J, Wang HL (2017). Graft-Free Maxillary Sinus Floor Elevation: A Systematic Review and Meta-Analysis. J Periodontol.

[17] Nkenke E, Stelzle F (2009). Clinical outcomes of sinus floor augmentation for implant placement using autogenous bone or bone substitutes: a systematic review. Clin Oral Implants Res.

[18] Marx RE, Carlson ER, Eichstaedt RM, Schimmele SR, Strauss JE, Georgeff KR (1998). Platelet-rich plasma: Growth factor enhancement for bone grafts. Oral Surg Oral Med Oral Pathol Oral Radiol Endod.

[19] Choukroun J, Diss A, Simonpieri A, Girard MO, Schoeffler C, Dohan SL (2006). Platelet-rich fibrin (PRF): a second-generation platelet concentrate. Part IV: clinical effects on tissue healing. Oral Surg Oral Med Oral Pathol Oral Radiol Endod.

[20] Lemos CA, Mello CC, dos Santos DM, Verri FR, Goiato MC, Pellizzer EP (2016). Effects of platelet-rich plasma in association with bone grafts in maxillary sinus augmentation: a systematic review and meta-analysis. Int J Oral Maxillofac Surg.

[21] Dragonas P, Katsaros T, Avila-Ortiz G, Chambrone L, Schiavo JH, Palaiologou A (2019). Effects of leukocyte-platelet-rich fibrin (L-PRF) in different intraoral bone grafting procedures: a systematic review. Int J Oral Maxillofac Surg.

[22] Dragonas P, Schiavo JH, Avila-Ortiz G, Palaiologou A, Katsaros T (2019). Plasma rich in growth factors (PRGF) in intraoral bone grafting procedures: A systematic review. J Craniomaxillofac Surg.

[23] Torres J, Tamimi F, Martinez PP, Alkhraisat MH, Linares R, Hernandez G (2009). Effect of platelet-rich plasma on sinus lifting: a randomized-controlled clinical trial. J Clin Periodontol.

[24] Sendyk DI, Chrcanovic BR, Albrektsson T, Wennerberg A, Zindel Deboni MC (2017). Does Surgical Experience Influence Implant Survival Rate? A Systematic Review and Meta-Analysis. Int J Prosthodont.

[25] Felice P, Pistilli R, Piattelli M, Soardi E, Barausse C, Esposito M (2014). 1-stage versus 2-stage lateral sinus lift procedures: 1-year post-loading results of a multicentre randomised controlled trial. Eur J Oral Implantol.

[26] Balleri P, Cozzolino A, Ghelli L, Momicchioli G, Varriale A (2002). Stability measurements of osseointegrated implants using Osstell in partially edentulous jaws after 1 year of loading: a pilot study. Clin Implant Dent Relat Res.

[27] Al-Moraissi E, Elsharkawy A, Abotaleb B, Alkebsi K, Al-Motwakel H (2018). Does intraoperative perforation of Schneiderian membrane during sinus lift surgery causes an increased the risk of implants failure?: A systematic review and meta regression analysis. Clin Implant Dent Relat Res.

[28] Taschieri S, Corbella S, Del Fabbro M (2012). Use of plasma rich in growth factor for schneiderian membrane management during maxillary sinus augmentation procedure. J Oral Implantol.

[29] Taschieri S, Corbella S, Tsesis I, Del Fabbro M (2014). Impact of the use of plasma rich in growth factors (PRGF) on the quality of life of patients treated with endodontic surgery when a perforation of sinus membrane occurred. A comparative study. Oral Maxillofac Surg.

[30] Taschieri S, Del Fabbro M (2011). Postextraction osteotome sinus floor elevation technique using plasma-rich growth factors. Implant Dent.

